# Individuals With SARS-CoV-2 Infection During the First and Second Waves in Catalonia, Spain: Retrospective Observational Study Using Daily Updated Data

**DOI:** 10.2196/30006

**Published:** 2022-01-06

**Authors:** Lia Alves-Cabratosa, Marc Comas-Cufí, Jordi Blanch, Ruth Martí-Lluch, Anna Ponjoan, Antoni Castro-Guardiola, Abelardo Hurtado-Ganoza, Ana Pérez-Jaén, Maria Rexach-Fumaña, Delfi Faixedas-Brunsoms, Maria Angels Gispert-Ametller, Anna Guell-Cargol, Maria Rodriguez-Batista, Ferran Santaularia-Font, Ramon Orriols, Marc Bonnin-Vilaplana, Juan Carlos Calderón López, Gladis Sabater-Talaverano, Francesc Xavier Queralt Moles, Sara Rodriguez-Requejo, Esteve Avellana-Revuelta, Elisabet Balló, Ester Fages-Masmiquel, Josep-Maria Sirvent, Carol Lorencio, Josep Miquel Morales-Pedrosa, Patricia Ortiz-Ballujera, Rafel Ramos

**Affiliations:** 1 Vascular Health Research Group of Girona Institut Universitari per a la Recerca en Atenció Primària Jordi Gol i Gurina Girona Spain; 2 Institut d'Investigació Biomèdica de Girona Girona Spain; 3 Internal Medicine Department Hospital Universitari de Girona Doctor Josep Trueta Girona Spain; 4 Department of Medical Sciences University of Girona Girona Spain; 5 Technical Secretariat Institut Assistència Sanitària Girona Spain; 6 Technical Secretariat Hospital Universitari de Girona Doctor Josep Trueta Girona Spain; 7 Emergency Department Hospital Universitari de Girona Doctor Josep Trueta Girona Spain; 8 Pneumology Department Hospital Universitari de Girona Doctor Josep Trueta Girona Spain; 9 Pneumology Department Hospital Santa Caterina de Salt Salt Spain; 10 CIBER of Respiratory Diseases Barcelona Spain; 11 Territorial Clinical Laboratory of Girona Parc Hospitalari Martí i Julià Salt Spain; 12 Atenció Primària Institut Català de la Salut Girona Spain; 13 Atenció Primària Institut d’Assistència Sanitària Girona Spain; 14 Intensive Care Department Hospital Universitari de Girona Doctor Josep Trueta Girona Spain

**Keywords:** epidemiology, SARS-CoV-2, COVID-19, timeline, comparison, pandemic, waves, population characteristics

## Abstract

**Background:**

A description of individuals with SARS-CoV-2 infection comparing the first and second waves could help adapt health services to manage this highly transmissible infection.

**Objective:**

We aimed to describe the epidemiology of individuals with suspected SARS-CoV-2 infection, and the characteristics of patients with a positive test comparing the first and second waves in Catalonia, Spain.

**Methods:**

This study had 2 stages. First, we analyzed daily updated data on SARS-CoV-2 infection in individuals from Girona (Catalonia). Second, we compared 2 retrospective cohorts of patients with a positive reverse-transcription polymerase chain reaction or rapid antigen test for SARS-CoV-2. The severity of patients with a positive test was defined by their admission to hospital, admission to intermediate respiratory care, admission to the intensive care unit, or death. The first wave was from March 1, 2020, to June 24, 2020, and the second wave was from June 25, 2020, to December 8, 2020.

**Results:**

The numbers of tests and cases were lower in the first wave than in the second wave (26,096 tests and 3140 cases in the first wave versus 140,332 tests and 11,800 cases in the second wave), but the percentage of positive results was higher in the first wave than in the second wave (12.0% versus 8.4%). Among individuals with a positive diagnostic test, 818 needed hospitalization in the first wave and 680 in the second; however, the percentage of hospitalized individuals was higher in the first wave than in the second wave (26.1% versus 5.8%). The group that was not admitted to hospital included older people and those with a higher percentage of comorbidities in the first wave, whereas the characteristics of the groups admitted to hospital were more alike.

**Conclusions:**

Screening systems for SARS-CoV-2 infection were scarce during the first wave, but were more adequate during the second wave, reflecting the usefulness of surveillance systems to detect a high number of asymptomatic infected individuals and their contacts, to help control this pandemic. The characteristics of individuals with SARS-CoV-2 infection in the first and second waves differed substantially; individuals in the first wave were older and had a worse health condition.

## Introduction

Since the first case of pneumonia caused by SARS-CoV-2 in December 2019, the pandemic struck the world with, probably, one of the most challenging outbreaks in the 21st century [1]. Nearly 90 million confirmed cases and nearly 2 million COVID-19–related deaths have occurred on all continents until January 11, 2021, as reported by the World Health Organization [2].

The first cases in Europe were detected in Italy and spread throughout the continent before societies realized the severity of the situation [3,4]. Health systems were suddenly burdened with individuals infected by this highly transmissible new disease, to the point of collapse in certain countries [5]. Strict lockdown measures were applied in most countries to decrease the number of cases and ensure adequate care for patients in critical condition [6]. These measures had a certain effectiveness, and the first COVID-19 wave faded away during the summer in Europe [7], only to give way to a second wave shortly after, with the easing of restrictions and presumably the initiation of the school term [8,9], although later reports questioned this [10-12], as well as the transfer of social life into indoor spaces [13]. The steady second increase of cases in Europe was initially evident in Spain from where it spread again, although this time at a slower pace, even within the Spanish regions [14]. After all, health systems had a short period to organize their structure if a second wave hit in the autumn, as was the case.

The arrival of the pandemic caught the health systems quite unaware and unprepared, and uncertainty had a synergic effect with the lack of knowledge about the new virus, the infection, and the disease [15-17]. As it spread, at the assistance level, the optimal actions to be taken were unclear [18]; at the management level, administrations had to adapt primary care and hospital health services; and at the informative level, the sources were neither prepared nor connected enough, and did not have methods to obtain reliable and complete data on SARS-CoV-2 infection [15,16]. Information systems on SARS-CoV-2 infection had to be built from scratch during the first wave and refined during the second wave.

Although much has been learnt about the virus and its transmissibility, many gaps in knowledge remain, including the comparison of the first wave and the entire second wave, which has received limited attention [19,20], and the consideration of individuals with various degrees of severity. Inquiry into such differences would improve our understanding of the effectiveness of the applied measures, and thus, it would help plan and improve the optimal public health strategies to tackle or at least alleviate the consequences of this infection. The evidence suggests that the context plays an important role in the presentation and spread of this infection [7,21]. Indeed, contributing factors and their weights may vary due to climatic conditions, government actions, culture, and behavior of the population, or could differ in patients attended in primary care settings and in hospitals [7,21]. At the time the study was conducted, Catalonia was facing the end of the second wave and foreseeing the possibility of the initiation of a third wave in the subsequent months [22]. A detailed epidemiological framework by country was recommended to consider the conditions for deployment of massive testing within the strategies to control this epidemic [22]. Accordingly, this study aimed to describe and compare the first and second waves of the SARS-CoV-2 epidemic in Catalonia (Spain). Particularly, we sought to report the daily counts, incidences, and numbers of hospitalized patients with this infection, and to compare the characteristics of cases in the first and second waves considering various degrees of severity.

## Methods

### Overview

This study was structured in 2 stages. First, in the general population, we examined the number of positive SARS-CoV-2 tests in each wave. Second, within the population with a positive test, we compared the characteristics of 2 retrospective cohorts, 1 for each wave. The first wave lasted from March 1, 2020, to June 24, 2020, and the second from June 25, 2020, to December 8, 2020.

### Analysis of the General Population

Enrollment included individuals from the province of Girona (Catalonia, Northern Spain), within the area of influence of Hospital Universitari de Girona Doctor Josep Trueta and Parc Hospitalari Martí i Julià from Salt (Girona).

For each wave, we counted the number of individuals with corresponding test results and the number of tests per diagnosis. On a daily basis, we tallied the number of individuals with a positive test from the general population, the daily empiric reproduction number at day 7 (ρ7; the empiric reproduction number is related to the reproduction number [23]), and the incidence rate of positive cases at 14 days. Pseudonymized data for these analyses were obtained from the primary care and hospital records.

### Comparison of Cohorts of Individuals With a Positive SARS-CoV-2 Test Result

The cohorts included individuals with a confirmed SARS-CoV-2 infection whose episode was closed, hereinafter also referred to as cases. Confirmed SARS-CoV-2 infection was defined by a positive test result, either using real-time reverse transcription polymerase chain reaction (RT-PCR) for SARS-CoV-2 [24] (requiring a cycle threshold under 39 as per laboratory standards in the daily routine of the 2 hospitals included in this study) or using a rapid antigen test [25-27]. The index date was the date of the positive test result, except where there was a COVID-19–related registry in the primary care center within 7 days before the positive test result, in which case the index date was the date of the visit instead. An episode was followed up to 30 days after a positive test result in the primary care records, if there was no record of hospital discharge; if there was a record, it was considered up until the time of discharge. For cases defined from the primary care records, death was considered if it occurred up until 30 days after a positive diagnostic test; for cases defined from hospital records, death was considered up until the time of discharge. Data records were obtained up to January 8, 2021.

For each wave, we characterized the cases (individuals with confirmed SARS-CoV-2 infection) using pseudonymized data registered in clinical health records from primary care. We considered the following variables up to the index date: age, sex, vascular risk factors (smoking, high alcohol consumption, obesity, diabetes mellitus, dyslipidemia, and hypertension), other comorbidities (atrial fibrillation, heart failure, ischemic heart disease, peripheral arterial disease, cerebrovascular disease, chronic obstructive pulmonary disease, asthma, sleep apnea, chronic kidney disease, malignant neoplasms, dementia, and depression), and treatment with acetylsalicylic acid. We also recorded previous influenza and pneumococcal vaccination, and calculated the Charlson index for every participant [28]. The Charlson index is a validated method to classify comorbidity, weighting the amount and severity of comorbid diseases in an integrated score that predicts 1-year mortality risk [29,30].

Censoring was applied at the time of closing the case. The highest degree of severity at censoring was the outcome. It was defined by admission to hospital, or lack of it, and department of admission (for admitted participants). Outcomes were considered by increasing severity as follows: mild infection (not admitted to a hospital), admitted to a conventional hospital (neither in intermediate respiratory care [IRC] nor in the intensive care unit [ICU]), admitted to IRC (ie, requiring noninvasive ventilation), admitted to the ICU (ie, requiring invasive ventilation), or death. Allocation of participants to the hospital departments was determined from pseudonymized inpatient administrative data, whereas allocation as mild infection (not admitted to hospital) was determined from pseudonymized hospital emergency records and from the primary health records.

For each wave, we estimated the cumulative incidence of the outcomes (degrees of severity) at 30 days. We also counted the total and daily numbers of individuals in hospital within cases (individuals with confirmed SARS-CoV-2 infection). For each degree of severity (outcome), baseline characteristics described cases in the first and second waves using the mean (SD) for continuous variables, and the cumulative number (percentage) for categorical variables; comparison of these characteristics was carried out using the Student *t* test for continuous variables and the Fisher exact test for categorical variables. The level of significance was set at .05. We also calculated the absolute differences of the means (95% CIs) for continuous variables and the odds ratios (ORs) (95% CIs) for categorical variables in the second wave with respect to the first. All analyses were performed using R software (version 4.0.3; R Foundation for Statistical Computing) [31].

## Results

### Overview

**Figure 1:**
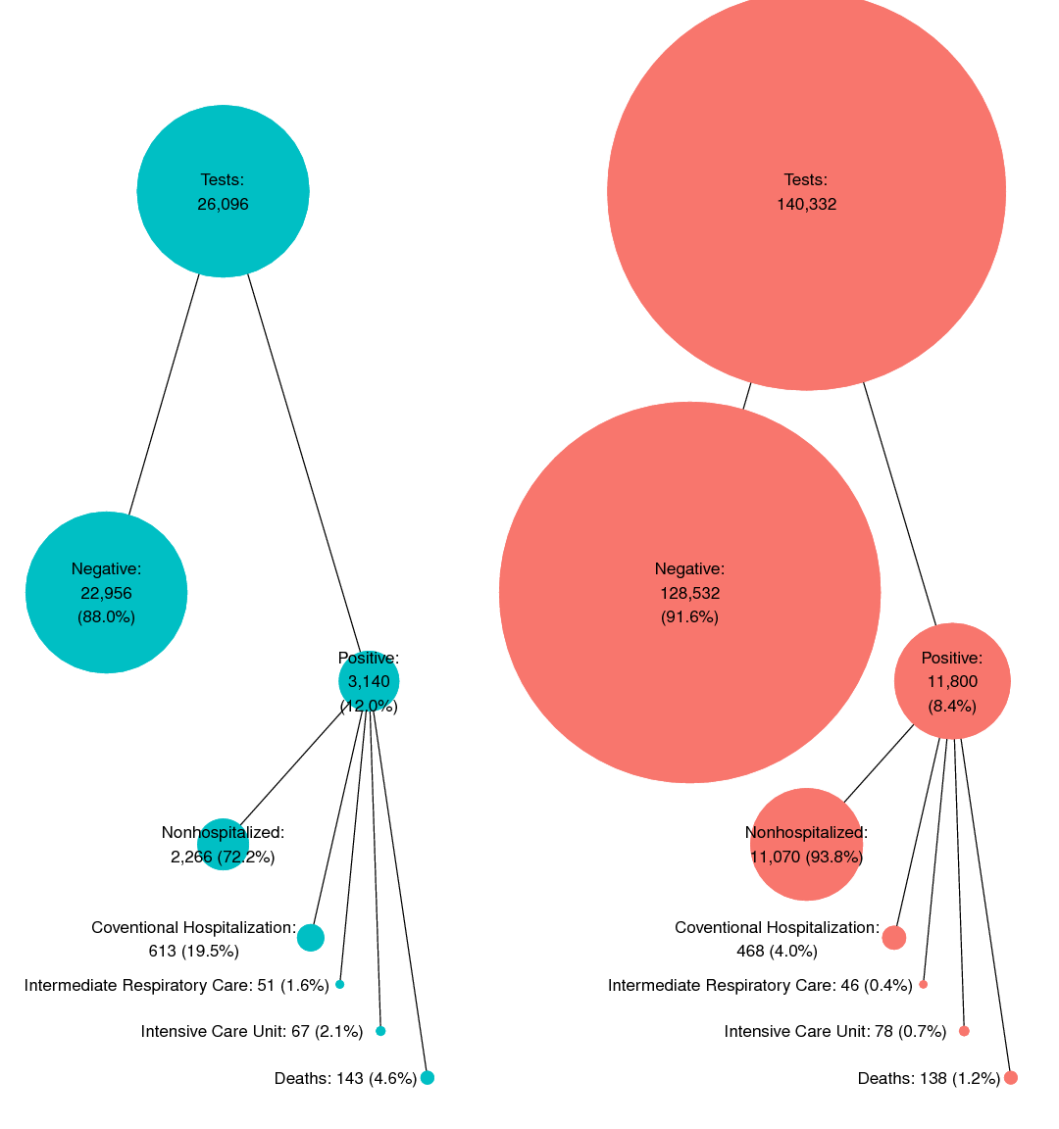
Comparison of the number and percentage of suspected and confirmed cases in the first (from March 1, 2020, to June 24, 2020) and second (from June 25, 2020, to December 8, 2020) SARS-CoV-2 waves in Girona (Catalonia).

Figure 1 provides a general overview of the 2 stages in this study. On the one hand, it shows the counts of positive tests in the general population; on the other hand, it shows the number of individuals for each outcome among those with a positive test.

### Analysis of the General Population

Total counts showed that the first wave had much lower numbers of positive cases (over 3000) than the second (nearly 12,000), but had a higher percentage of positive tests with respect to all suspected individuals (12.0% in the first wave versus 8.4% in the second) (Figure 1). The number of tests per case was 8.3 in the first wave (a total of 26,096 tests and 3140 cases) and 11.9 in the second wave (a total of 140,332 tests and 11,800 cases).

**Figure 2:**
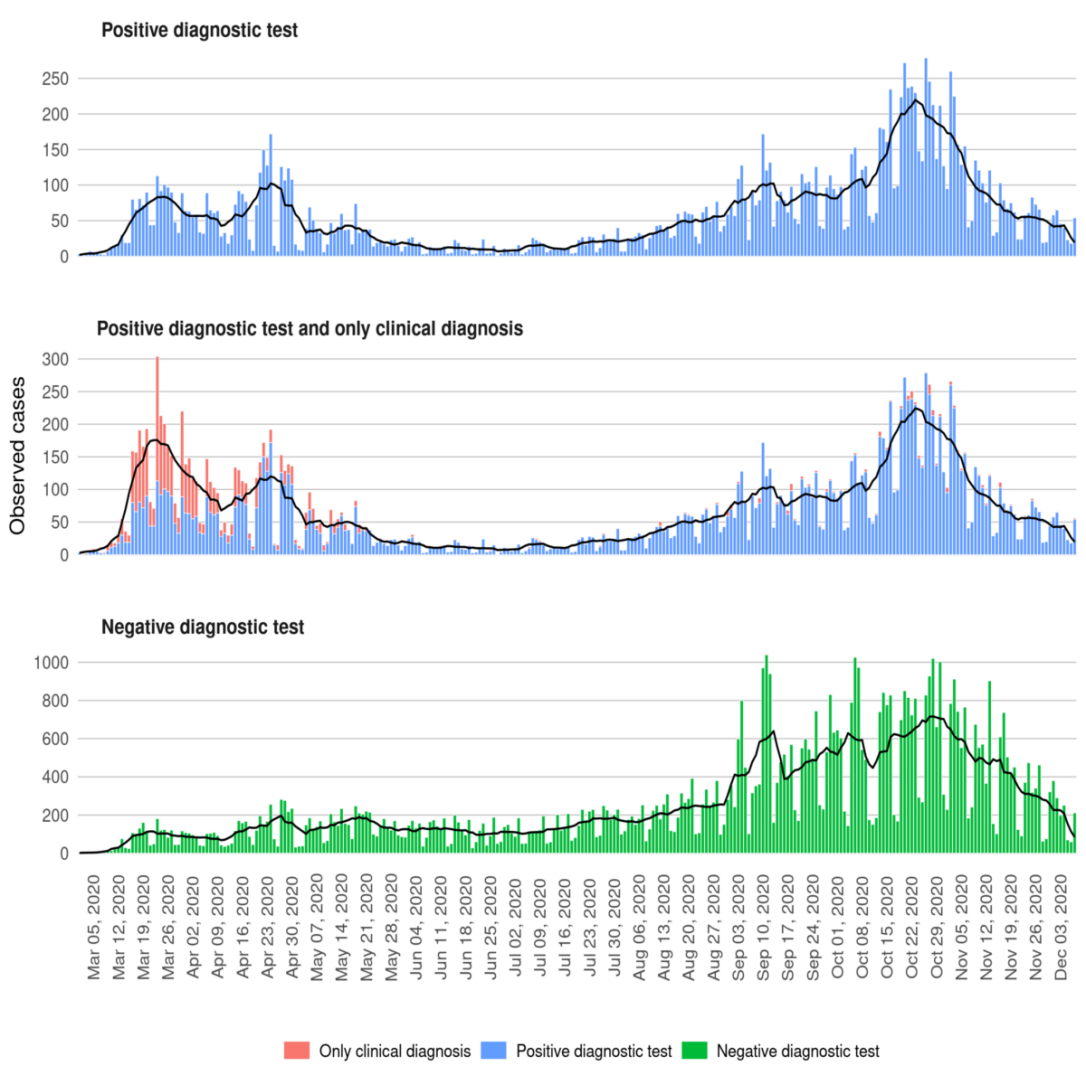
Daily number of individuals with a positive and/or negative SARS-CoV-2 test in Girona (Catalonia) from March 1, 2020, to December 8, 2020.

Two waves could be clearly distinguished in the timeline of COVID-19 cases. The first wave of the overall population (hospitalized and nonhospitalized) showed an increase of cases in March (Figure 2). Then, the number of cases decreased until the beginning of summer (at the end of June), when a slow increasing trend appeared again (Figure 2). The second wave was longer, and many more positive cases were detected in that period (nearly 4-fold) (Figure 1). However, when we considered an additional group of possible cases, that is, individuals with no confirmatory test but with symptoms compatible with COVID-19 (indicated as only clinical diagnosis in Figure 2), the situation became more even (Figure 2). Figure 2 also shows that the number of daily negative diagnostic test results was much higher in the second wave.

**Figure 3:**
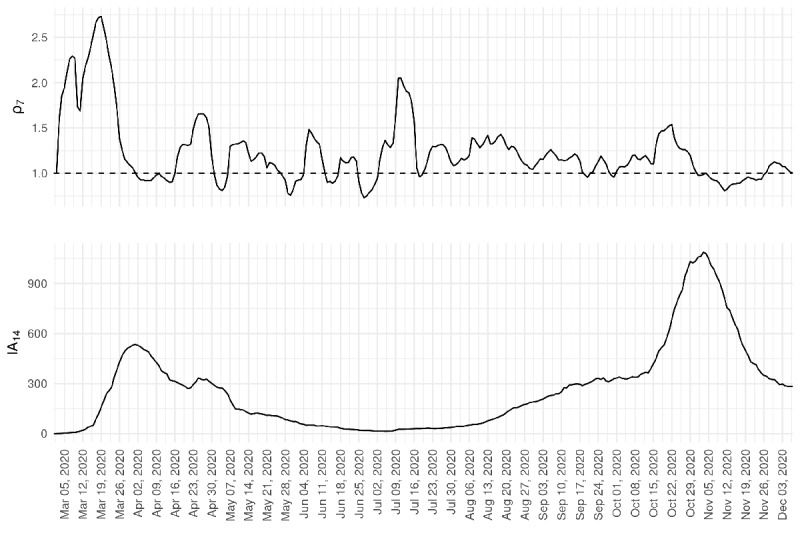
Daily evolution of the empiric reproduction number (7 days; ρ7) and cumulative incidence rate (14 days; IA14) of positive cases in Girona (Catalonia), from March 1, 2020, to December 8, 2020.

SARS-CoV-2 transmission in the community was also monitored with the cumulative incidence rate of SARS-CoV-2 infection at 14 days and with the transmission rate at 7 days, indicated by the empiric reproduction number (ρ7) (Figure 3). At the beginning of the first wave, the ρ7 value increased, followed by an increase in the incidence rate. Social distancing and ultimately strict lockdown led to a drop in the ρ7 value; when it was under 1, the incidence started to decrease. The decrease went on as far as the ρ7 value was predominantly under 1. However, at the end of June, the ρ7 reached a value over 1 and remained there, which led to a slow but constant increase in the incidence rate and subsequently to the second wave.

### Comparison of Cohorts of Individuals With a Positive SARS-CoV-2 Test

Figure 1 shows the cumulative incidence at 30 days for each outcome within each wave, with respect to all individuals with a positive test result (cases). The first wave contained a lower percentage of individuals with mild SARS-CoV-2 infection (nonhospitalized) and higher percentages of individuals who were in a conventional hospital, who were admitted to IRC, who were admitted to the ICU, and who passed away (including in-hospital and out-of-hospital deaths).

**Figure 4:**
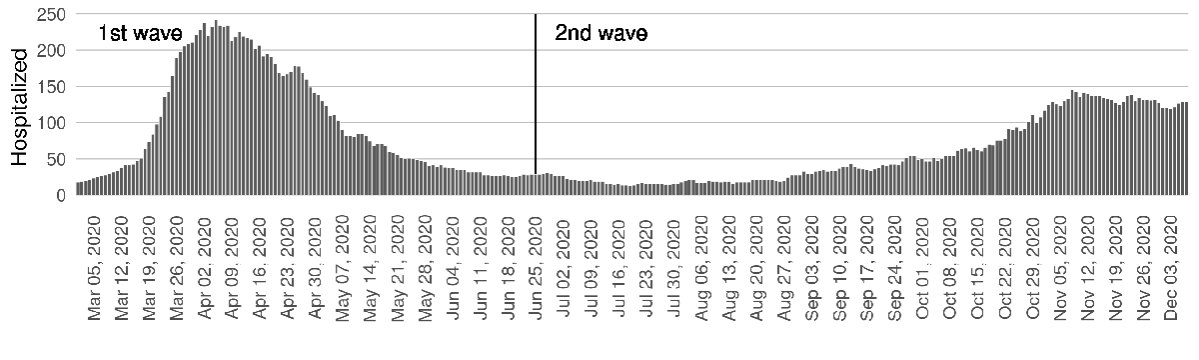
Daily number of individuals with a positive SARS-CoV-2 test in hospital over time (from March 1, 2020, to December 8, 2020) in Girona (Catalonia).

Hospitalized cases amounted to 818 out of 3140 cases in the first wave and 680 out of 11,800 cases in the second, with cumulative incidences at 30 days of 26.1% and 5.8%, respectively. During the first wave, 613 patients (a cumulative incidence at 30 days of 74.9%, with respect to all hospitalized) were in a conventional hospital, 51 (6.2%) were in IRC, 67 (8.2%) were in the ICU, and 87 (10.6%) passed away. The corresponding figures among hospitalized cases during the second wave were 468 (68.8%), 46 (6.8%), 78 (11.5%), and 88 (12.9%), respectively. The daily number of individuals in hospital showed a much steeper increase during the first wave than the second, the initiation of which was more progressive (Figure 4).

Comparison of the baseline characteristics (individuals with a positive test [cases]) showed that individuals with mild SARS-CoV-2 infection (no hospital admission) were almost 10 years older in the first wave (*P*<.001) (Table 1). The absolute difference in the mean age between the second wave and the first supported statistical significance (Multimedia Appendix 1); the absolute difference was −8.67 (95% CI −9.71 to −7.63). Regarding other degrees of severity, the mean ages of individuals with conventional hospitalization and individuals admitted to the ICU were slightly higher in the second wave (*P*=.04 and *P*=.02, respectively) (Tables 1 and 2). The 95% CI of the absolute differences supported statistical significance; they were 2.5 (95% CI 0.15-4.85) and 5.15 (95% CI 0.66-9.64), respectively. As for the rest of the population characteristics, the percentage of individuals with other comorbidities and risk factors in the first wave was mostly higher than in the second, in the group with no hospital admission (Table 1), with significant *P* values. These results were supported by ORs under 1 and with significant 95% CIs (Multimedia Appendix 1). Characteristics in the rest of the groups (hospitalized) were similar in the first and second waves, with few exceptions (Tables 1 and 2). In the second wave, the group admitted to a conventional hospital had a higher percentage of individuals with dyslipidemia, hypertension, and cerebrovascular disease, and receiving acetylsalicylic acid (Table 1). These results were supported by ORs over 1 and with significant 95% CIs (Multimedia Appendix 1). The group admitted to IRC included a higher percentage of individuals receiving acetylsalicylic acid in the second wave (Table 2), and the OR comparing the second wave with the first wave was 3.01 with significance and a 95% CI of 1.63-5.77. The group admitted to the ICU had a higher percentage of individuals with diabetes and a higher Charlson index in the second wave (Table 2), with significant *P* values and significance of the 95% CIs of the ORs and the absolute differences. Finally, the group of deceased individuals had a higher percentage of patients with atrial fibrillation, previous pneumococcal vaccination, and treatment with acetylsalicylic acid in the second wave (Table 2). The *P* values for the differences and the 95% CIs of the ORs supported statistical significance.

**Table 1:** Comparison of the baseline characteristics of individuals with a positive SARS-CoV-2 test from Girona (Catalonia) in the first (March 1, 2020, to June 24, 2020) and second (June 25, 2020, to December 8, 2020) SARS-CoV-2 waves in the no admission and admission but no intermediate respiratory care or intensive care unit groups.

Variable	No admission				Admission but no IRC^a^ or ICU^b^		
	1st wave (n=2266)	2nd wave (n=11,070)	*P* value	1st wave (n=613)		2nd wave (n=468)	*P* value
Age, mean (SD)	54.5 (22.3)	45.8 (26.3)	<.001	58.6 (18.9)		61.1 (20.0)	.04
Men, n (%)	703 (31.0)	5223 (47.2)	<.001	300 (48.9)		235 (50.2)	.71
Smoker, n (%)	393 (23.9)	1910 (26.5)	.10	92 (17.9)		54 (13.7)	.24
Exsmoker, n (%)	140 (8.5)	603 (8.4)	.10	74 (14.4)		61 (15.5)	.24
Alcohol consumption of high risk, n (%)	35 (1.5)	115 (1.0)	.048	19 (3.1)		15 (3.2)	.99
Obesity, n (%)	538 (23.7)	2404 (21.7)	.16	248 (40.5)		205 (43.8)	.23
Diabetes, n (%)	194 (8.6)	575 (5.2)	<.001	117 (19.1)		110 (23.5)	.08
Dyslipidemia, n (%)	420 (18.5)	1192 (10.8)	<.001	169 (27.6)		158 (33.8)	.03
Hypertension, n (%)	574 (25.3)	1475 (13.3)	<.001	220 (35.9)		215 (45.9)	.001
Atrial fibrillation, n (%)	102 (4.5)	145 (1.3)	<.001	44 (7.2)		33 (7.1)	.99
Heart failure, n (%)	63 (2.8)	51 (0.5)	<.001	23 (3.8)		19 (4.1)	.87
Ischemic heart disease, n (%)	64 (2.8)	146 (1.3)	<.001	38 (6.2)		37 (7.9)	.28
PAD^c^, n (%)	52 (2.3)	91 (0.8)	<.001	24 (3.9)		24 (5.1)	.37
Cerebrovascular disease, n (%)	52 (2.3)	84 (0.8)	<.001	15 (2.4)		29 (6.2)	.003
COPD^d^, n (%)	65 (2.9)	137 (1.2)	<.001	44 (7.2)		33 (7.1)	.99
Asthma, n (%)	132 (5.8)	539 (4.9)	.06	42 (6.9)		24 (5.1)	.25
Sleep apnea, n (%)	56 (2.5)	210 (1.9)	.08	33 (5.4)		33 (7.1)	.30
Chronic kidney disease, n (%)	167 (7.4)	217 (2.0)	<.001	61 (10.0)		65 (13.9)	.05
Malignant neoplasms, n (%)	175 (7.7)	358 (3.2)	<.001	69 (11.3)		52 (11.1)	.99
Dementia, n (%)	232 (10.2)	208 (1.9)	<.001	44 (7.2)		35 (7.5)	.91
Depression, n (%)	199 (8.8)	530 (4.8)	<.001	73 (11.9)		44 (9.4)	.20
Previous flu vaccination, n (%)	598 (26.4)	1254 (11.3)	<.001	183 (29.9)		142 (30.3)	.89
Previous pneumococcus vaccination, n (%)	533 (23.5)	1739 (15.7)	<.001	199 (32.5)		179 (38.2)	.05
ASA^e^, n (%)	57 (2.5)	190 (1.7)	.01	17 (2.8)		37 (7.9)	<.001
Charlson index, mean (SD)	2.3 (2.0)	2.0 (1.8)	<.001	2.8 (2.3)		2.8 (2.2)	.86

^a^IRC: intermediate respiratory care.

^b^ICU: intensive care unit.

^c^PAD: peripheral arterial disease.

^d^COPD: chronic obstructive pulmonary disease.

^e^ASA: acetylsalicylic acid.

**Table 2:** Comparison of the baseline characteristics of individuals with a positive SARS-CoV-2 test from Girona (Catalonia) in the first (March 1, 2020, to June 24, 2020) and second (June 25, 2020, to December 8, 2020) SARS-CoV-2 waves in the admission to intermediate respiratory care, admission to the intensive care unit, and deceased groups.

Variable	Admission to IRC^a^				Admission to the ICU^b^				Deceased		
	1st wave (n=51)	2nd wave (n=46)	*P* value	1st wave (n=67)		2nd wave (n=78)	*P* value	1st wave (n=143)		2nd wave (n=138)	*P* value
Age, mean (SD)	64.6 (14.7)	61.7 (15.4)	.35	56.2 (13.1)		61.3 (14.2)	.02	81.0 (12.4)		81.7 (11.3)	.61
Men, n (%)	34 (66.7)	30 (65.2)	.99	48 (71.6)		63 (80.8)	.24	73 (51.0)		69 (50.0)	.90
Smoker, n (%)	5 (10.4)	6 (14.3)	.83	6 (10.7)		6 (9.0)	.49	17 (13.8)		17 (14.2)	.46
Exsmoker, n (%)	8 (16.7)	6 (14.3)	.83	10 (17.9)		18 (26.9)	.49	13 (10.6)		19 (15.8)	.46
Alcohol consumption of high risk, n (%)	0 (0.0)	0 (0.0)	N/A^c^	2 (3.0)		3 (3.8)	.99	7 (4.9)		6 (4.3)	.99
Obesity, n (%)	30 (58.8)	25 (54.3)	.15	33 (49.3)		39 (50.0)	.79	50 (35.0)		53 (38.4)	.50
Diabetes, n (%)	16 (31.4)	7 (15.2)	.09	9 (13.4)		25 (32.1)	.01	47 (32.9)		51 (37.0)	.53
Dyslipidemia, n (%)	17 (33.3)	17 (37.0)	.83	22 (32.8)		27 (34.6)	.86	55 (38.5)		65 (47.1)	.15
Hypertension, n (%)	24 (47.1)	24 (52.2)	.69	24 (35.8)		39 (50.0)	.09	101 (70.6)		106 (76.8)	.28
Atrial fibrillation, n (%)	9 (17.6)	3 (6.5)	.13	1 (1.5)		1 (1.3)	.99	18 (12.6)		35 (25.4)	.009
Heart failure, n (%)	4 (7.8)	2 (4.3)	.68	0 (0.0)		1 (1.3)	.99	11 (7.7)		17 (12.3)	.23
Ischemic heart disease, n (%)	8 (15.7)	4 (8.7)	.36	3 (4.5)		8 (10.3)	.22	14 (9.8)		21 (15.2)	.21
PAD^d^, n (%)	1 (2.0)	1 (2.2)	.99	3 (4.5)		3 (3.8)	.99	11 (7.7)		6 (4.3)	.32
Cerebrovascular disease, n (%)	2 (3.9)	1 (2.2)	.99	2 (3.0)		1 (1.3)	.60	11 (7.7)		12 (8.7)	.83
COPD^e^, n (%)	10 (19.6)	6 (13.0)	.42	2 (3.0)		4 (5.1)	.69	15 (10.5)		19 (13.8)	.47
Asthma, n (%)	3 (5.9)	4 (8.7)	.70	2 (3.0)		3 (3.8)	.99	4 (2.8)		11 (8.0)	.06
Sleep apnea, n (%)	9 (17.6)	3 (6.5)	.13	5 (7.5)		6 (7.7)	.99	7 (4.9)		7 (5.1)	.99
Chronic kidney disease, n (%)	9 (17.6)	7 (15.2)	.79	3 (4.5)		6 (7.7)	.51	42 (29.4)		46 (33.3)	.52
Malignant neoplasms, n (%)	9 (17.6)	4 (8.7)	.24	7 (10.4)		13 (16.7)	.34	51 (35.7)		42 (30.4)	.38
Dementia, n (%)	2 (3.9)	1 (2.2)	.99	0 (0.0)		2 (2.6)	.50	46 (32.2)		38 (27.5)	.43
Depression, n (%)	7 (13.7)	3 (6.5)	.32	4 (6.0)		6 (7.7)	.75	22 (15.4)		26 (18.8)	.53
Previous flu vaccination, n (%)	19 (37.3)	17 (37.0)	.99	14 (20.9)		15 (19.2)	.84	84 (58.7)		79 (57.2)	.81
Previous pneumococcus vaccination, n (%)	24 (47.1)	16 (34.8)	.30	17 (25.4)		26 (33.3)	.36	85 (59.4)		107 (77.5)	.001
ASA^f^, n (%)	0 (0.0)	4 (8.7)	.047	3 (4.5)		7 (9.0)	.34	12 (8.4)		24 (17.4)	.03
Charlson index, mean (SD)	2.8 (2.4)	1.9 (1.2)	.07	1.7 (1.1)		2.7 (2.7)	.03	3.1 (2.3)		3.5 (2.7)	.23

^a^IRC: intermediate respiratory care.

^b^ICU: intensive care unit.

^c^N/A: not applicable.

^d^PAD: peripheral arterial disease.

^e^COPD: chronic obstructive pulmonary disease.

^f^ASA: acetylsalicylic acid.

## Discussion

### Principal Findings

We compared the epidemiology and characteristics of individuals with SARS-CoV-2 infection in the first and second waves in Catalonia. The first wave struck more suddenly, and although SARS-CoV-2–positive individuals were less numerous, the percentage with respect to all suspected individuals was higher than in the second wave. Moreover, individuals with a positive diagnostic test were healthier in the second wave, as indicated by the lower proportion of individuals who required hospitalization (26.1% in the first wave versus 5.8% in the second) and the lower percentage of patients with comorbidities among nonhospitalized patients. However, these lower percentages might also be attributed to the younger age of the population in the second wave, because younger individuals tend to have a better health condition. Once in hospital, the differences in age and comorbidities between the first and second waves were much less prominent.

During the first wave, no screening for the general population was performed, simply because there was no time to organize screenings and tests were not available for everyone. In March and April 2020, RT-PCR tests were performed for patients admitted to the hospital and for health workers, and up to early June, screenings were directed at old people in nursing homes, centers for disabled individuals, supervised flats, and penitentiaries. These screenings represented one-third of all PCR tests carried out during the first wave (ie, PCR tests were prioritized for the most vulnerable populations). However, if we consider the number of clinically diagnosed cases in the first wave (individuals who were considered to have COVID-19 based on signs and symptoms, but in whom no diagnosis test was performed), the number of individuals with COVID-19 appears similar. Even conceding that the infection spread was just starting during the period included in the first wave, it is likely that a large number of asymptomatic cases were unnoticed in that wave. This idea is supported by previous reports [32] and is coherent with our results. Figure 2 and Figure 4 show that hospitalized cases were more numerous and the number increased more abruptly in the first wave than in the second wave (Figure 4), but the number of daily overall cases detected with diagnostic tests was much lower in the first wave (Figure 2).

In the second wave, surveillance and health systems were more organized and proactive, especially in areas where the transmission rate increased, which allowed a huge amount of screening tests to be carried out. This volume of tests during the second wave would explain the much higher number of positive cases (almost 4-fold) than in the first wave. The lower percentage of positive cases in the second wave shows the efforts and success of the screening systems to find, test, and isolate contacts when needed. This is another crucial aspect in the epidemiology comparing the first and second waves in this pandemic (the means to diagnose the infection, the consideration of a person as a case, the availability of diagnostic tests, and the capacity of the surveillance systems to organize screenings and preventive measures at a large scale) [33].

In hospitals, the situation was also very different during the 2 waves. The first wave arrived so suddenly that the system collapsed, and the criteria to allocate and treat patients according to severity kept changing and were different from the second wave. During the second wave, the population, especially vulnerable individuals, knew how to protect themselves, which smoothened the increase of cases, and thus, the situation in hospitals was tense but the system did not collapse. The criteria to allocate and treat patients were more established, and health professionals could be more proactive to admit and treat patients with milder forms of the disease.

Within hospitalized patients, the second wave included a higher percentage of individuals with certain conditions in the group of patients with conventional hospitalization (dyslipidemia, hypertension, cerebrovascular disease, or treatment with acetylsalicylic acid), those with admission to the ICU (diabetes), or those who passed away (atrial fibrillation, pneumococcus vaccination, and treatment with acetylsalicylic acid). This could be partly explained because of a slightly higher age average. Finally, the second wave lasted longer than the first, which resulted in a fairly similar total number of patients in IRC, those in intensive care, and those who passed away in both waves.

### Strengths and Limitations

We had access to daily updated and reliable data that could be structured for analysis up to a date that included the second wave. Moreover, we could assess all individuals with a diagnostic test for SARS-CoV-2 (ie, with a negative or positive result), which allowed a complete description of the situation, since a high number of positive mild cases could be, as the case actually was, due to an increase in the number of tests performed. However, we acknowledge that in February and March 2020, clinical diagnosis or the definition of close contacts was determined according to epidemiological criteria from countries that first reported COVID-19 cases (China [34] and Italy [35]); thus, many patients who must have been positive were not identified as such, and some close contacts were overlooked. Additionally, antigen tests were not available in the first wave and were only available in the second wave. In this second wave, the tests were performed in certain situations, like screening in schools or in symptomatic individuals, and the criteria to apply them changed to adapt and avoid too much pressure on the health systems. We decided to include them in the analysis to be able to account for all individuals who tested positive and appraise the performance of the screening.

### Comparison With Prior Work

A Letter to the Editor on an analysis from Japan reported higher pressure on the health system, higher proportion of individuals with comorbidities, and older mean age in the first wave, in line with our results. However, they could not include data to complete the second wave, and thus, there is a possibility that future findings differ from their results at the time of publication [19]. Nevertheless, comparison of results in Japan and the south of Europe remains of high interest. Indeed, preparedness for the pandemic differed between countries before [36] and during the spread of the pandemic. Some countries had some time to equip themselves for the second wave, but they could not adapt readily enough to it, with subsequent burden on the health system and thus the population [32]. Further analyses that compare the first and second waves in other countries would be very useful to determine expected common characteristics and differences. A couple of previous reports characterized the first wave in Spain, as in April and August 2020 [37,38]. The authors of a report from the Working Group for the Surveillance and Control of COVID-19 observed a much higher percentage of hospitalized patients among individuals who tested positive in a diagnostic test when compared with our study (45% versus 11%), which could be explained by the definition of a case. They considered a person as a case if they had symptoms of severe acute respiratory infection and had travelled to COVID-19–affected areas or had epidemiological links with COVID-19 laboratory-confirmed cases [37]. Finally, an analysis of the first wave in Catalonia studied data from the primary care setting to compare the characteristics of individuals with and without COVID-19, and deceased and living patients with COVID-19; our results in the first wave for nonhospitalized individuals and for deceased patients are comparable to the findings in this study [38].

### Conclusions

Screening systems for SARS-CoV-2 infection were scarce during the first wave, but were more adequate during the second wave, reflecting the usefulness of surveillance systems to detect a high number of asymptomatic infected individuals and their contacts, to help control this pandemic. Individuals infected by SARS-CoV-2 differed substantially during the first and second waves in Catalonia. Infected individuals were older and had more comorbidities in the first wave, and more of them needed hospitalization. Hospitals collapsed in the first wave, but tension was lower in the second wave, which contributed to better care for a broader spectrum of the population.

## Appendix

Multimedia Appendix 1 Absolute differences and odds ratios for the baseline characteristics of individuals with a positive SARS-CoV-2 test result from Girona (Catalonia) on comparing the second SARS-CoV-2 wave (June 25, 2020, to December 8, 2020) to the first wave (March 1, 2020, to June 24, 2020).

## Abbreviations

ρ7: empiric reproduction number at day 7
ICU: intensive care unit
IRC: intermediate respiratory care
OR: odds ratio
RT-PCR: reverse-transcription polymerase chain reaction
