# Supplementary material for: Individuals With SARS-CoV-2 Infection During the First and Second Waves in Catalonia, Spain: Retrospective Observational Study Using Daily Updated Data
Source: JMIR Public Health Surveill. 2022 Jan 6;8(1):e30006. doi: 10.2196/30006 (PMC8734611; doi:10.2196/30006)
Supplement: Multimedia Appendix 1 [file publichealth_v8i1e30006_app1.docx]

**Multimedia Appendix 1.** Absolute differences and odds ratios for the baseline characteristics of individuals with a positive SARS-CoV-2 test result from Girona (Catalonia) on comparing the second SARS-CoV-2 wave (June 25, 2020, to December 8, 2020) to the first wave (March 1, 2020, to June 24, 2020).

|  | **^No admission^** | **^Admission no IRC or ICU^** | **^Admission IRC^** | **^Admission ICU^** | **^Deceased^** |
| --- | --- | --- | --- | --- | --- |
| **^Wave^** | **^OR^** | | | | |
| **^Age^** | ^-8.67 (95%IC: -9.71; -7.63)^ | ^2.50 (95%IC: .15; 4.85)^ | ^-2.85 (95%IC: -8.94; 3.24)^ | ^5.15 (95%IC: .66; 9.64)^ | ^.71 (95%IC: -2.07; 3.49)^ |
| **^Men^** | ^1.99 (95%IC: 1.80; 2.19)^ | ^1.05 (95%IC: .82; 1.35)^ | ^.94 (95%IC: .37; 2.37)^ | ^1.66 (95%IC: .71; 3.90)^ | ^.96 (95%IC: .58; 1.57)^ |
| **^Alcohol consumption of high risk^** | ^.67 (95%IC: .45; 1.01)^ | ^1.04 (95%IC: .48; 2.18)^ | ^-^ | ^1.30 (95%IC: .14; 15.98)^ | ^.88 (95%IC: .24; 3.16)^ |
| **^Obesity^** | ^.89 (95%IC: .80; .99)^ | ^1.15 (95%IC: .89; 1.47)^ | ^.83 (95%IC: .35; 2.01)^ | ^1.03 (95%IC: .51; 2.08)^ | ^1.16 (95%IC: .69; 1.94)^ |
| **^Diabetes^** | ^.59 (95%IC: .49; .70)^ | ^1.30 (95%IC: .96; 1.77)^ | ^.40 (95%IC: .12; 1.16)^ | ^3.02 (95%IC: 1.23; 8.04)^ | ^1.20 (95%IC: .71; 2.02)^ |
| **^Dyslipidemia^** | ^.53 (95%IC: .47; .60)^ | ^1.34 (95%IC: 1.02; 1.75)^ | ^1.17 (95%IC: .47; 2.94)^ | ^1.08 (95%IC: .51; 2.30)^ | ^1.42 (95%IC: .86; 2.35)^ |
| **^Hypertension^** | ^.45 (95%IC: .41; .51)^ | ^1.52 (95%IC: 1.18; 1.96)^ | ^1.22 (95%IC: .51; 2.94)^ | ^1.78 (95%IC: .87; 3.70)^ | ^1.38 (95%IC: .78; 2.44)^ |
| **^Atrial fibrillation^** | ^.28 (95%IC: .22; .37)^ | ^.98 (95%IC: .59; 1.61)^ | ^.33 (95%IC: .05; 1.44)^ | ^.86 (95%IC: .01; 68.26)^ | ^2.35 (95%IC: 1.21; 4.69)^ |
| **^Heart Failure^** | ^.16 (95%IC: .11; .24)^ | ^1.09 (95%IC: .55; 2.11)^ | ^.54 (95%IC: .05; 3.96)^ | ^-^ | ^1.68 (95%IC: .71; 4.15)^ |
| **^Ischemic heart disease^** | ^.46 (95%IC: .34; .63)^ | ^1.30 (95%IC: .79; 2.14)^ | ^.52 (95%IC: .11; 2.10)^ | ^2.42 (95%IC: .55; 14.80)^ | ^1.65 (95%IC: .76; 3.68)^ |
| **^PAD^** | ^.35 (95%IC: .25; .51)^ | ^1.33 (95%IC: .71; 2.47)^ | ^1.11 (95%IC: .01; 88.88)^ | ^.85 (95%IC: .11; 6.60)^ | ^.55 (95%IC: .16; 1.67)^ |
| **^Cerebrovascular disease^** | ^.33 (95%IC: .23; .47)^ | ^2.63 (95%IC: 1.35; 5.35)^ | ^.55 (95%IC: .01; 1.86)^ | ^.42 (95%IC: .01; 8.33)^ | ^1.14 (95%IC: .44; 2.97)^ |
| **^COPD^** | ^.42 (95%IC: .31; .58)^ | ^.98 (95%IC: .59; 1.61)^ | ^.62 (95%IC: .17; 2.09)^ | ^1.75 (95%IC: .24; 19.94)^ | ^1.36 (95%IC: .62; 3.02)^ |
| **^Asthma^** | ^.83 (95%IC: .68; 1.01)^ | ^.74 (95%IC: .42; 1.26)^ | ^1.52 (95%IC: .24; 1.95)^ | ^1.30 (95%IC: .14; 15.98)^ | ^3.00 (95%IC: .86; 13.24)^ |
| **^Sleep apnea^** | ^.76 (95%IC: .56; 1.05)^ | ^1.33 (95%IC: .78; 2.27)^ | ^.33 (95%IC: .05; 1.44)^ | ^1.03 (95%IC: .25; 4.50)^ | ^1.04 (95%IC: .30; 3.57)^ |
| **^Chronic kidney disease^** | ^.25 (95%IC: .20; .31)^ | ^1.46 (95%IC: .99; 2.16)^ | ^.84 (95%IC: .24; 2.81)^ | ^1.77 (95%IC: .36; 11.39)^ | ^1.20 (95%IC: .70; 2.06)^ |
| **^Malignant neoplasms^** | ^.40 (95%IC: .33; .48)^ | ^.99 (95%IC: .66; 1.47)^ | ^.45 (95%IC: .09; 1.76)^ | ^1.71 (95%IC: .59; 5.41)^ | ^.79 (95%IC: .46; 1.34)^ |
| **^Dementia^** | ^.17 (95%IC: .14; .20)^ | ^1.05 (95%IC: .64; 1.70)^ | ^.55 (95%IC: .01; 1.86)^ | ^-^ | ^.80 (95%IC: .46; 1.38)^ |
| **^Depression^** | ^.52 (95%IC: .44; .62)^ | ^.77 (95%IC: .50; 1.16)^ | ^.44 (95%IC: .07; 2.09)^ | ^1.31 (95%IC: .30; 6.61)^ | ^1.28 (95%IC: .65; 2.51)^ |
| **^Previous flu vaccination^** | ^.36 (95%IC: .32; .40)^ | ^1.02 (95%IC: .78; 1.34)^ | ^.99 (95%IC: .40; 2.44)^ | ^.90 (95%IC: .37; 2.22)^ | ^.94 (95%IC: .57; 1.55)^ |
| **^Previous pneumococcus vaccination^** | ^.61 (95%IC: .54; .68)^ | ^1.29 (95%IC: .99; 1.67)^ | ^.60 (95%IC: .24; 1.47)^ | ^1.47 (95%IC: .67; 3.26)^ | ^2.35 (95%IC: 1.36; 4.12)^ |
| **^ASA^** | ^.68 (95%IC: .50; .93)^ | ^3.01 (95%IC: 1.63; 5.77)^ | ^-^ | ^2.09 (95%IC: .45; 13.07)^ | ^2.29 (95%IC: 1.05; 5.27)^ |
| **^Charlson index^** | ^-.38 (95%IC: -0.54; -0.22)^ | ^.03 (95%IC: -0.34; .41)^ | ^-.88 (95%IC: -1.83; .08)^ | ^1.01 (95%IC: .13; 1.90)^ | ^.39 (95%IC: -0.25; 1.02)^ |

Values are absolute differences (95% CI), or odds ratios (95% CI).

ASA indicates acetylsalicylic acid; COPD, chronic obstructive pulmonary disease; IRC, intermediate respiratory care; ICU, intensive care unit; OR, odds ratio; PAD, peripheral arterial disease.
